# Exploring the development of face recognition across childhood via logistic mixed-effects modelling of the standardised Cambridge Face Memory Test

**DOI:** 10.3758/s13428-025-02629-y

**Published:** 2025-03-10

**Authors:** Louise Ewing, Nadja Althaus, Emily K. Farran, Michael Papasavva, Inês Mares, Marie L. Smith

**Affiliations:** 1 School of Psychology, University of East Anglia, Norwich Research Park, Norwich, NR4 7TJ UK; 2 School of Psychology, University of Surrey, Guildford, GU2 7XH UK; 3 Centre for Genomics and Child Health, Blizard Institute, Queen Mary, University of London, London, UK; 4 William James Centre for Research, ISPA – Instituto Universitário, Lisboa, Portugal; 5 School of Psychological Science, Birkbeck College, University of London, London, UK

**Keywords:** Face recognition, Face memory, Development, Children, Gender, Multilevel methods, CFMT

## Abstract

Individual differences in face identity recognition abilities are present across the lifespan but require developmentally differentiated methods of assessment. Here, we examine the empirical validity of a widely used face identity recognition measure, the Cambridge Face Memory Test for Children (CFMT-C). Logistic mixed-effects modelling of a large data set (607 children, 5–12 years) replicates and extends the findings of the only previous normative study of the CFMT-C (Croydon et al., *Neuropsychologia*, *62*, 60–67, 2014). This novel, analytical approach enables us to take into account sources of variability typically overlooked in a classical analysis. We consider variability introduced by the task, alongside variability across children, to provide the first comprehensive characterisation of the interactive effects of factors inherent to participants (e.g. age, gender, and ethnicity), and the test (stage: face learning, simple recognition, harder recognition) on face memory performance. In line with past findings, we clearly observed age-related improvement in the task. Additionally, and for the first time, we report that this developmental effect is significantly more pronounced in the later, harder stages of the task; that there is an effect of gender, with females having better performance; and that consideration of participant ethnicity or testing context did not alter the best fitting model of these data. These results highlight the value of applying multilevel statistical models to characterise the factors driving performance variability, providing evidence of the divergence in recognition abilities across genders and confirming the stability of the CFMT-C in assessing face recognition abilities across variable experimental contexts and with diverse participant groups.

**Supplementary information:**

The online version contains supplementary material available at 10.3758/s13428-025-02629-y.

## Introduction

Identity recognition is crucial for successful everyday functioning, and is unsurprisingly one of the most studied aspects of face expertise. Researchers and clinical neuropsychologists have developed a number of standardised tests to characterise these abilities (e.g. Benton Face Recognition Test, Benton & Van Allen, 1968; Warrington Recognition Memory Test, Warrington, 1984). Typically, these measures have been designed with young adults in mind, and most of the behavioural and neuroimaging studies investigating the underpinnings of expertise in this domain focus on these cohorts. Yet perceptual expertise with faces changes considerably across developmental time (e.g. Germine et al., 2011) and the individual differences that are of interest to researchers during adulthood (Wilmer, 2017), are also prevalent during childhood (Bennetts et al., 2017) and later adulthood (Boutet & Meinhardt-Injac, 2021). Clear characterisation of the development of this vital social function and its determinants is thus critically contingent upon reliable and sensitive psychometric tools that are appropriate to use with individuals across the lifespan.

The most widely used standardised test of face recognition ability is the Cambridge Face Memory Test (CFMT, Duchaine & Nakayama, 2006). It is a targeted measure of short-term, unfamiliar face memory developed for adults (see also Arrington et al., 2022; Dennett et al., 2012; Kho et al., 2023; McKone et al., 2011; McKone et al., 2017 for examples of parallel forms of the test). The task was designed to be sensitive to individual differences in face recognition ability in neurotypical and neurodiverse populations, including in those with clinically impaired abilities (subsequent extensions further facilitate sensitivity to performance differences in the upmost ‘super-recogniser’ range of ability, e.g. CFMT+ Russell et al., 2009). Within the face processing literature, performance on the CFMT (percent correct recognition accuracy) has been studied in its own right, e.g. when exploring differences in face expertise from adolescence to old age (12 to 70 years, Germine et al., 2011). It is also used extensively as an independent behavioural benchmark for other metrics, e.g. when probing associations with behavioural and neural processes predicted to functionally contribute to face selective expertise (e.g. Mares et al., 2023; McGugin et al., 2016; Richler et al., 2011; Rhodes et al., 2014; Wilmer et al., 2012). Evaluations to date support its strong psychometric properties (Bowles et al., 2009; Cho et al., 2015; Herzmann et al., 2008; Wilmer et al., 2012) validating its wide use as a valid and reliable measure to study face recognition ability in adults.

Briefly, the CMFT introduces participants to a set of six novel/unfamiliar adult face identities to be learned and then recognised alongside foils in a series of three alternative forced-choice (3AFC) decisions. It comprises three ‘task stages’ that increase progressively in difficulty, each presenting participants with different challenges for face recognition. Stage 1 test images are identical to the studied target faces. Stage 2 test images must be recognised in novel viewpoints and/or lighting conditions, which precludes a pictorial or ‘image-based’ processing strategy. Stage 3 test faces must be identified in novel views *and* with the addition of Gaussian visual noise that particularly obscures featural information and encourages reliance on specialised face processing mechanisms, e.g. holistic coding.

Despite being relatively brief and easy to complete for young and older adults (simple instructions, computerised administration, 10–15-min duration) the standard measure was not designed to be used with children. It presents a substantial cognitive challenge for them, which may particularly encourage the use of atypical/immature strategies (e.g. feature-based strategies, see O’Hearn et al., 2010). As a result, two alternate versions have been designed for children, each taking a slightly different approach to its developmental adaptation. The CFMT for Children (CFMT-C; Croydon et al., 2014) retains the original adult face stimuli, but requires participants of all ages (5–12 years) to learn five identities (rather than six) and employs a simplified 2AFC trial format across all three task stages. The CFMT-Kids (CFMT-K; Dalrymple et al., 2014) rather opts to use child faces in line with its target sample and varies the number of faces to be learned based on participant age: four identities for younger children (9 years and under), and six for older children. While both approaches have successfully characterised face processing variability in developmental samples, the CFMT-C is of particular interest due to its methodological equivalence over a wider age range of middle childhood, and suitability for adult populations with impaired cognitive abilities given that it retains adult faces (see Farran et al., 2020).

A single detailed investigation of the psychometric properties of the CFMT-C supports its reliability and validity as a test of face identity recognition across middle childhood (5 to 12 years, Croydon et al., 2014). In a large group of primary and secondary school-aged neurotypical children, the measure yielded a spread of scores that avoided floor and ceiling effects, and was sensitive to age-related gains across the targeted age range. The CFMT-C also characterised difficulties in face identity recognition in a separate group of autistic children. Notably, however, Croydon et al. (2014) did not identify any significant differences between the performance of boys and girls on the CFMT-C. This finding is surprising given that gender differences in face identity recognition are reported consistently in adults, i.e. an advantage in female participants relative to males (including on the CFMT, Wilmer et al., 2012), as well as in children (see Herlitz & Lovén, 2013 for a review). Moreover, this female advantage is reported to be of similar magnitude across child, adolescent, and adult samples (meta-analysis of studies with participants aged 4–11, 12–17, and 18–53 years, Herlitz & Lovén, 2013), supporting an early developmental origin. Further investigation of the CFMT-C is warranted to establish if the lack of gender differences in identity recognition during development replicates in a larger sample and with more comprehensive analysis.

We also note that the ethnicity of participants was not reported nor analysed with regards to CFMT-C scores in Croydon et al. (2014). Given that exclusively white faces are presented during the CFMT-C, expected differences in ethnicity within their sample (primary and secondary schools in England have diverse student populations) may have had an unknown impact on children’s reported recognition abilities (e.g. ‘other ethnicity effects’, see Meissner & Brigham, 2001 for a review). Incorporating the potential inherent variability introduced by factors such as participant ethnicity in a single analytic model should provide better characterisation of the underlying data.

Finally, in several recent studies with adult populations, researchers have questioned whether all three stages of the CFMT are equally informative in quantifying individual face recognition ability. This work has benchmarked ‘empirical utility’ by focusing on the extent to which scores from each section benefit the reliable and efficient identification of prosopagnosia (individuals with very low levels of expertise). Studies report the measure’s sensitivity and specificity to prosopagnosia is similar when including vs. omitting scores from this final, most challenging stage, due likely to the poorer performance of control as well as prosopagnosic subjects when presented with face stimuli in visual noise (Corrow et al., 2018; Murray & Bate, 2020). Thus, an abridged version might constitute an equally effective – but crucially more efficient – instrument for identifying very poor performing individuals (see also Cho et al., 2015). Whether this is similarly the case for the CFMT-C’s sensitivity to developmental effects is of empirical value to establish, since relatively poorer recognition abilities in young compared to older children could be masked in the third stage if performance is attenuated broadly. In their study, Croydon et al. (2014) reported a significant main effect of stage (with performance decreasing progressively in each later stage, as expected) and no interaction with age. This finding suggests there was no change in the age-related differences in performance observed in each experimental stage. We revisit this question in the current study, to explore if age-related differences in the relative utility of the three task stages might emerge when applying a particularly sensitive analytical approach.

Here, we set out to provide a comprehensive investigation of the potentially interacting effects of participant age (5–12 years), participant gender, participant ethnicity, and stage of test (1: initial face learning, 2: simple recognition, 3: harder recognition) on task performance, alongside replicating the basic effects of the suitability of the CFMT-C as a measure for exploring development in face recognition performance with participant age. For the first time, we are also able to consider the potential contribution of testing context to performance; having run the CFMT-C in a number of different studies that were conducted across variable locations. We will establish whether modelling of task performance data benefits from distinguishing between highly controlled lab-based testing settings vs more ‘noisy’ community testing settings, such as in schools and museums.

Importantly, we go beyond the standard linear analysis used in the previous normative study of the CFMT-C by Croydon and colleagues (2014), which does not take into account the fact that individual participants typically exhibit different intercepts (a measure of their own ability), and that the effect of task stage may also differ in magnitude across participants. In other words, each individual will have higher/lower general performance compared to the mean of their age group (intercept), but the extent to which this performance is impacted by the different stages may also vary across individuals. There is no a priori way of establishing whether the addition of noise, for instance, will result in a similar drop in performance across all participants, even at the same age.

Linear mixed-effects models have recently become an established technique to provide fine-grained insight into manual response data (e.g. Barr et al., 2013) including on face recognition tasks (e.g. Arrington et al., 2022; Childs et al., 2021), precisely because they allow, by means of structured random effects, to estimate participant-specific, stimulus-specific, and stage-specific parameters instead of treating the entire data set as though the effect is the same across all participants and stages. Using a suitable random effects structure effectively means attributing more of the variability in the data to these potentially relevant factors instead of treating any deviance from the mean as ‘error’. As a consequence, the final estimate of the fixed effects can be more precise. We therefore chose to apply this more complex modelling approach to our analysis, using binomial logistic mixed-effects models, which are suitable for accuracy (binomial) data (Dixon, 2008)^1^.

## Methods

### Participants

Responses on the CFMT-C were collected as part of seven different data collection efforts with non-overlapping samples between 2014 and 2021 (in part previously published in Ewing et al., 2018, 2022; Farran et al., 2020; Mares et al., 2020). The task was a constituent element of a number of different studies run by our research group based in a large and diverse UK city. These studies varied considerably in the specifics of the testing team (though all individuals were trained to conduct developmental testing by the same individual), as well as the setting: science museum, laboratory, ‘holiday camp’ program, school. Using an integrative data analysis approach (Curran & Hussong, 2009) these data were compiled to create a large sample of responses. Individuals who achieved below-chance accuracy (i.e. average accuracy <.5) on Stage 1 trials were excluded (*N*= 6;* N *= 1 at 5 years,* N *= 1 at 6 years,* N *= 1 at 7 years,* N *= 1 at 8 years,* N *= 2 at 9 years), since this suggests either a high level of inattention or a failure to understand the instructions, given the simplicity of this part of the task (see below).

Each study had ethical approval (Reference Codes: 161721, 131464/5/6, 161756/7) and included a similar consent procedure: children provided verbal assent and parents provided written consent. The total number of participants was 607. Children were aged 5 to 12 years, 377 identifying as white and 117 identifying with other ethnicities, which were combined into a single category^2^, 358 girls and 249 boys, see Table 1 for details (and Supplementary Materials Table 1 for the sample broken up into their separate study cohorts/testing contexts). Because face identity recognition ability is known to be impaired in individuals with some neurodevelopmental conditions, in lab-based experiments, we routinely pre-screen participants for neurotypical development. However, in community testing settings, our policy is to be as inclusive as possible. Therefore, we only excluded data from any individuals who themselves or a family member/caregiver disclosed having a diagnosed neurodevelopmental condition at the time of consent. For the same inclusivity reasons, and to maximise the large sample sizes required for well-powered analyses, attempts were not made to match males and females by variables such as ethnicity.

**Table 1:** Mean performance (% correct accuracy) per age and gender on the different stages of the CFMT-C

		Stage 1 (Intro)		Stage 2 (No noise)		Stage 3 (Noise)		Total	
Age (years)	Gender	M	SD	M	SD	M	SD	M	SD
5	F	82.7	12.5	64.8	17.1	64.0	13.0	69.0	11.1
	M	94.7	8.7	63.2	16.1	57.0	28.0	69.0	13.6
6	F	88.4	11.8	75.2	13.6	66.0	15.7	75.4	11.4
	M	90.6	11.1	70.5	15.1	61.0	14.9	72.4	11.7
7	F	92.0	10.7	80.1	13.9	67.5	14.3	78.9	10.6
	M	90.0	10.2	74.2	13.3	62.0	14.2	74.1	9.6
8	F	93.8	7.7	80.1	12.0	70.7	11.3	80.4	9.0
	M	94.8	6.9	82.0	12.2	71.9	16.9	81.8	9.9
9	F	95.3	8.7	83.2	12.9	71.2	11.8	82.2	9.1
	M	94.1	9.0	76.8	15.0	66.4	12.1	77.7	9.2
10	F	96.4	5.7	85.3	11.1	74.1	15.1	84.3	9.2
	M	97.0	5.3	80.8	14.1	65.1	15.8	79.6	10.4
11	F	97.9	5.6	89.2	11.2	77.7	14.8	87.6	9.3
	M	96.1	6.4	81.6	12.2	68.1	15.0	80.7	10.2
12	F	99.2	2.3	79.8	21.9	76.3	18.8	83.4	14.4
	M	96.7	8.5	84.4	11.5	69.5	15.2	82.5	9.7

### Stimuli and procedure

Detailed methods information about the CFMT-C is available elsewhere (e.g. Croydon et al., 2014). Briefly, the task involves learning a set of five adult male face identities, and then recognizing these individuals when they are presented alongside foils in a series of 2AFC decisions. The task comprises three stages that progressively increase in difficulty. First, the target identities must be identified when presented alongside faces that are identical to the learned images (Stage 1, 15 trials), then from images that vary in viewpoint and/or lighting (Stage 2, 25 trials), and then from images that have been additionally obscured with Gaussian visual noise (Stage 3, 20 trials). The 60 test trials are presented in a fixed order.

In the current study, all participants completed the upright version of the CFMT-C in conjunction with other face and object processing measures that varied depending on the specific study for which they were recruited. The position of the CFMT-C in the testing battery sequence (e.g. first, second, last, etc) was not controlled across studies, nor were specific supervision arrangements: though the maximum child-to-experimenter ratio was 3:1. Additionally, all experimenters were trained in the administration of the task by the same individual which ensured consistency in style (e.g. positive rapport was always established before commencement, participant engagement was closely monitored and re-established if deemed to be wavering, and effort was encouraged and reinforced with enthusiastic praise).

## Results

Overall performance accuracy on the CFMT-C is typically calculated as percent correct, i.e. correct trials summed across all three task stages/total possible (= 60). For individual stages, performance is calculated as a percentage of correct trials out of the trials in that stage (Stage 1: total possible = 15, Stage 2: 25, Stage 3: 20).

### Ceiling effects

One sample *t* tests indicated that 5-year-olds were neither at chance nor ceiling for all Stages (all *p*s < .005), as were 6-, 7-, 8-, 9-, 10- and 11-year-olds (all *p*s < .001). Only the 12-year-olds in Stage 1 were at ceiling (*t*(25) = 1.66, *p* = .11), with their Stage 2 and 3 performances significantly below ceiling (*p*s < .001). Five-year-olds achieved a mean accuracy of 85.7% in Stage 1, 64.4% in Stage 2, and 62.3% in Stage 3 (69% overall). Twelve-year-olds’ average accuracy was 98.2% in Stage 1, 81.5% in Stage 2, and 73.7% in Stage 3 (83.1% overall). This is comparable to data presented by Croydon et al., although they did not find ceiling effects in the oldest group even for Stage 1.

### Linear regression and ANOVA

As a first step, we aimed to conduct an analysis identical to the one published by Croydon et al. (2014), i.e. to fit a simple regression line to the data with a fixed effect of age (without random effects or other fixed effects). This yielded an intercept of 79.5% accuracy and a slope of 1.837 meaning the model was defined as Y = 79.5 + 1.837 * age. This outcome is broadly comparable to Croydon et al., who reported Y = 51.89 + 2.78 * age. The main difference appears to be within the youngest age groups, who tended to obtain relatively higher scores in our experiments.

In parallel to Croydon et al., we then also conducted an ANOVA with factors age group (5, 6, 7, 8, 9, 10, 11, 12 years), gender (female, male), and stage (1, 2, 3). As expected, this analysis yielded a main effect of age (*F*(7, 591) = 10.88, *p* < .0001, η^2^ = .070 (for post hoc pairwise comparisons, see Table 2). In contrast to Croydon et al., we also observed a main effect of gender (*F*(1,591) = 6.668, *p* = .01, η^2^ = .007) with girls (M = 80.87, SD = 10.85) performing better than boys (M = 77.81, SD = 10.53). There was also a significant main effect of Stage (*F*(1.96, 1159.13) = 628.909, *p* < .0001, η^2^ = .305, Greenhouse–Geisser corrected). As we might expect, and consistent with Croydon et al. (2014), given the increasingly difficult task demands, performance was significantly superior in Stage 1 (M = 93.79, SD = 9.18) compared to Stage 2 (M = 79.84, SD = 14.3), which was also superior to Stage 3 (M = 68.69, SD = 14.97), all *t*s ≥ 13.0, all *p*s < .001. We observed a significant interaction of stage x gender (*F*(1.96, 1159.13) = 11.012, *p* = < .0001, η^2^ = .008, Greenhouse–Geisser), with females significantly outperforming males only in Stage 3 (*t*(591) = 3.81, *p* = .002, Bonferroni-corrected, all others *p* > .51). There was no stage x age interaction, *F*(13.73, 1159.13) = 1.470, *p* = .117, η^2^ = .007. We note that Croydon et al. (2014) report no interactions of stage with age or with gender. Finally, there was no interaction of age x gender (*p* = .281), and no three-way interaction of age, stage, and gender (*p* = .212).

**Table 2:** Pairwise post hoc comparisons of overall CFMT-C task performance for participant age (Bonferroni-corrected p values)

Age	6	7	8	9	10	11	12
5	1.0	0.346	**= .001**	**= .004**	**< .001**	**< .001**	**< .001**
6		1.0	**< .001**	**0.003**	**< .001**	**< .001**	**0.002**
7			**0.032**	0.132	**0.001**	**< .001**	**0.047**
8				1.0	1.0	1.0	1.0
9					1.0	0.136	1.0
10						1.0	1.0
11							1.0

Pairwise post hoc comparisons of overall CFMT-C task performance for participant age (Bonferroni-corrected *p* values)

### Mixed-effects modelling

For this more detailed analysis we fitted models to individual trial-level data/responses, which facilitates a greater degree of sensitivity to systematic variability present in the data. Binomial logistic mixed-effects models were fitted using the *lme4* package in R (Bates et al., 2015), and random intercepts for participants and trials/items were included in all models. Random slopes for participants were also included in all models with a fixed effect of stage. We performed our analysis in two steps, determining first in a series of model comparisons which random effects, fixed effects (age, gender, stage) or interactions improved model fit and which did not. In the second step, we inspected the best-fitting model that resulted from this procedure. This relatively conservative process of gradually increasing the complexity of the model ensures the inclusion in the final model of only those terms that are necessary in order to sufficiently capture the variability in the data. Essentially, for every fixed effect or interaction, the question is asked whether a model that includes this term provides a better explanation of the data than the previous model (without this term). If a specific fixed effect does not improve the model either when included as a main effect or included as part of an interaction, then that effect is not included in the final model because there is a simpler model that explains the data equally well. Goodness-of-fit is evaluated with chi square tests using the *lrtest* function from the *lmtest* package (Zeileis & Hothorn, 2002). Therefore, in this next section, **χ**^**2**^ statistics indicate the outcome of these comparisons, i.e. whether or not the addition of a fixed effect improves the model fit with significant scores indicating the value of an added effect.

Once the best-fitting model has been determined we report, given this model, which of the main effects or interactions are found to be significant.

#### Ethnicity

Because ethnicity information was missing for 113 participants, we first conducted a preliminary model comparison with only the 494 participants for whom ethnicity information was available (coded as white, *N* = 377, vs. all other ethnic groups combined, *N* = 117). As described above, this first (baseline) model included factors of age, gender, stage, and associated interactions. We confirmed that adding a main effect of ethnicity or any of the interactions involving ethnicity did not improve the model fit (all *p* > .22, cf. Supplementary Table 2). Given these results, we concluded that participants’ ethnicity did not play a significant role in participants’ performance on the CFMT, and proceeded with model comparisons using the full data set (i.e. including those participants with missing ethnicity information) without the factor ethnicity.

#### Model comparisons

The next step was a base model using all of the data (*N* = 607) including no fixed or random effects. Then we added random intercepts of trials/items and participants in order to assert that a model with those random effects is indeed a better fit than without, i.e. a multilevel approach is preferable. Following this, fixed effects of age, gender and stage were added in a stepwise fashion as well as their corresponding interactions.

Both, the addition of a random effect of trials/items on intercepts (**χ**^**2**^(1) = 3905.5, *p* < .0001), and the addition of a random effect of participants on intercepts (**χ**^**2**^(1) = 1458.3, *p* < .0001) improved the model fit, justifying a multilevel approach. We then added between-participants fixed effects of age (**χ**^**2**^(1) = 62.83, *p* < .0001) and gender (**χ**^**2**^(1) = 19.88, *p* = .0001), which also improved the model fit. However, the interaction of age x gender did not further improve the model (**χ**^**2**^(1) = 2.9452, *p* = .09).

By contrast, adding a main effect of stage did improve the model further (**χ**^**2**^(2) = 52.79, *p* < .0001), as did the addition of random slopes to take into account individual participants’ differences in the effect of this predictor (**χ**^**2**^(5) = 155.03, *p* < .0001). The interaction of age x stage improved the model further, **χ**^**2**^(2) = 51.41, *p* < .0001, but not the interaction of gender x stage or the three-way interaction between these factors (all *p* > .32).

All effects and interactions that did not improve the model further were removed (see Tables 3, 4, in Supplementary Materials for the revised model comparison statistics with only these effects). Finally, to address the possibility that the different experimental contexts in which these data were collected might have affected participants’ performance, we also tested whether the inclusion of a fixed between-subjects effect “Experiment” (with seven levels, see Supplementary Table 1) or any of its interactions with the remaining factors improved the model. This analysis (see Table 5 in Supplementary Materials) confirmed that the addition of the main effect did not improve the model fit, nor any of the interactions (all *p*s > .1), so this variable was not considered further.

We concluded that the final best-fitting model was then the one including fixed effects of age, gender, and stage and a two-way interaction of age x stage^3^. This model is summarised in Table 3.

**Table 3:** Estimated coefficients for best-fitting model (see Footnote 1)

	Estimate (accuracy)	Estimate (logit)	Std. Error	*p*
(Intercept)	.976	3.69	0.20	< .0001
Age	.007	0.36	0.04	< .0001
Gender (male)	– .009	– 0.31	0.06	< .0001
Stage (2)	– .110	– 1.83	0.24	< .0001
Stage (3)	– .237	– 2.65	0.25	< .0001
Age x stage (2)	– .005	– 0.19	0.04	< .0001
Age x stage (3)	– .007	– 0.27	0.04	< .0001

Estimates provided with respect to base levels Gender = female, Stage = 1/Intro. The variable age was centred at the mean age 8.5 years, and the estimate for the fixed effect age corresponds to the expected increment per year. The intercept indicates the model’s predicted accuracy at the mean age, 8.5 years. The estimates for fixed effects and interaction indicate the increment with regard to the intercept that is predicted for the corresponding combination of parameters (e.g. on average a difference of 1 year corresponds to a difference of .007 in accuracy, whereas for boys the average accuracy is .009 below girls)

### Best-fitting model

The best-fitting model confirms that all effects are present, i.e. fixed effects of age (*p* < .0001), gender (*p* < .0001) and stage (*p* < .0001) were significant, as was the interaction of age x stage (*p* < .0001). The model did not show a significantly better fit when including an age x gender, gender x stage or age x gender x stage interaction (see Table 3, Supplementary Material).

**Fig. 1:**
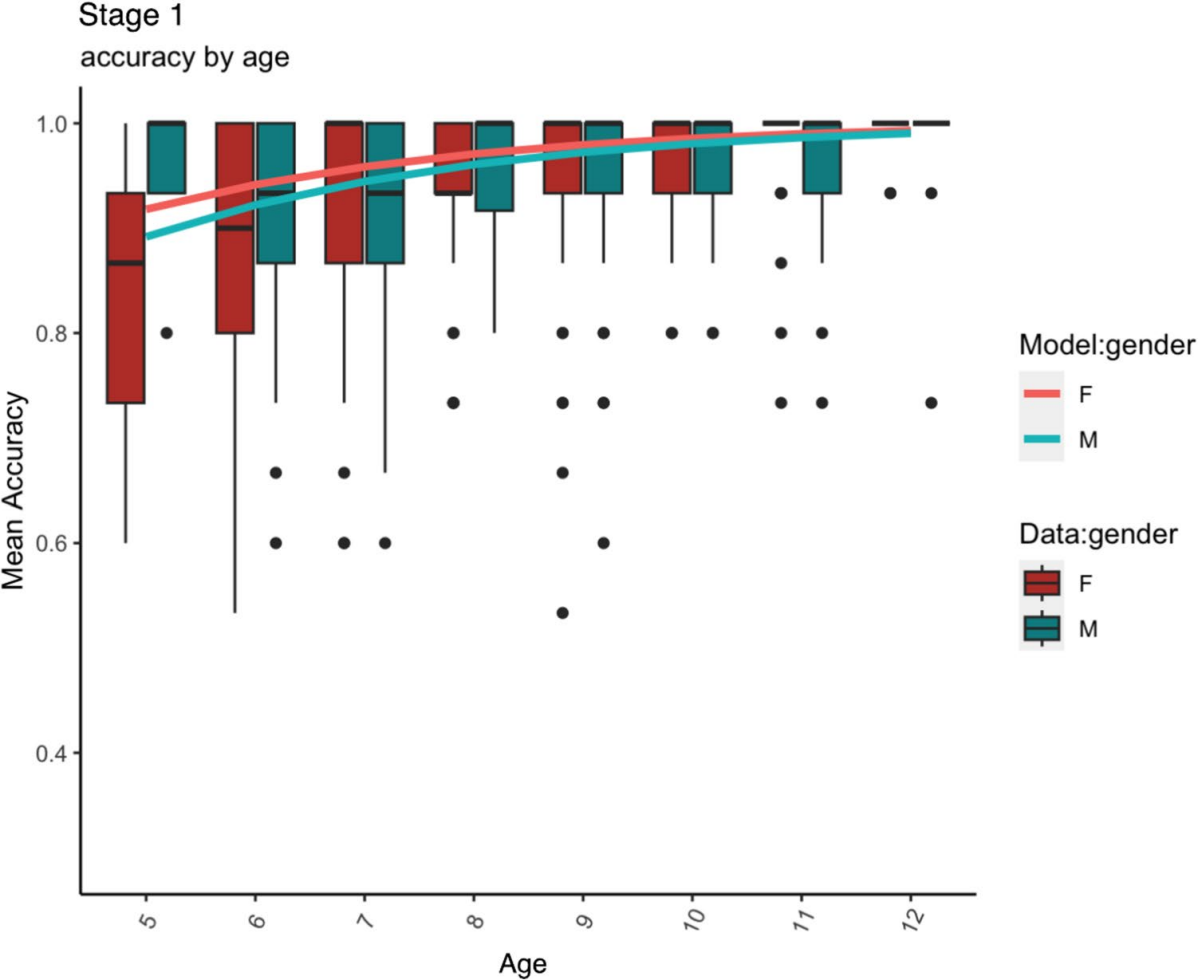
Stage 1 (Intro): Mean accuracy data (box plots: *horizontal black lines* indicate medians, the *box* shows the interquartile range and *whiskers* show largest/smallest values within 1.5 times interquartile range above 75^th^/below 25^th^ percentile, *black filled circles* show outliers) and model predictions (***line plots***)

**Fig. 2:**
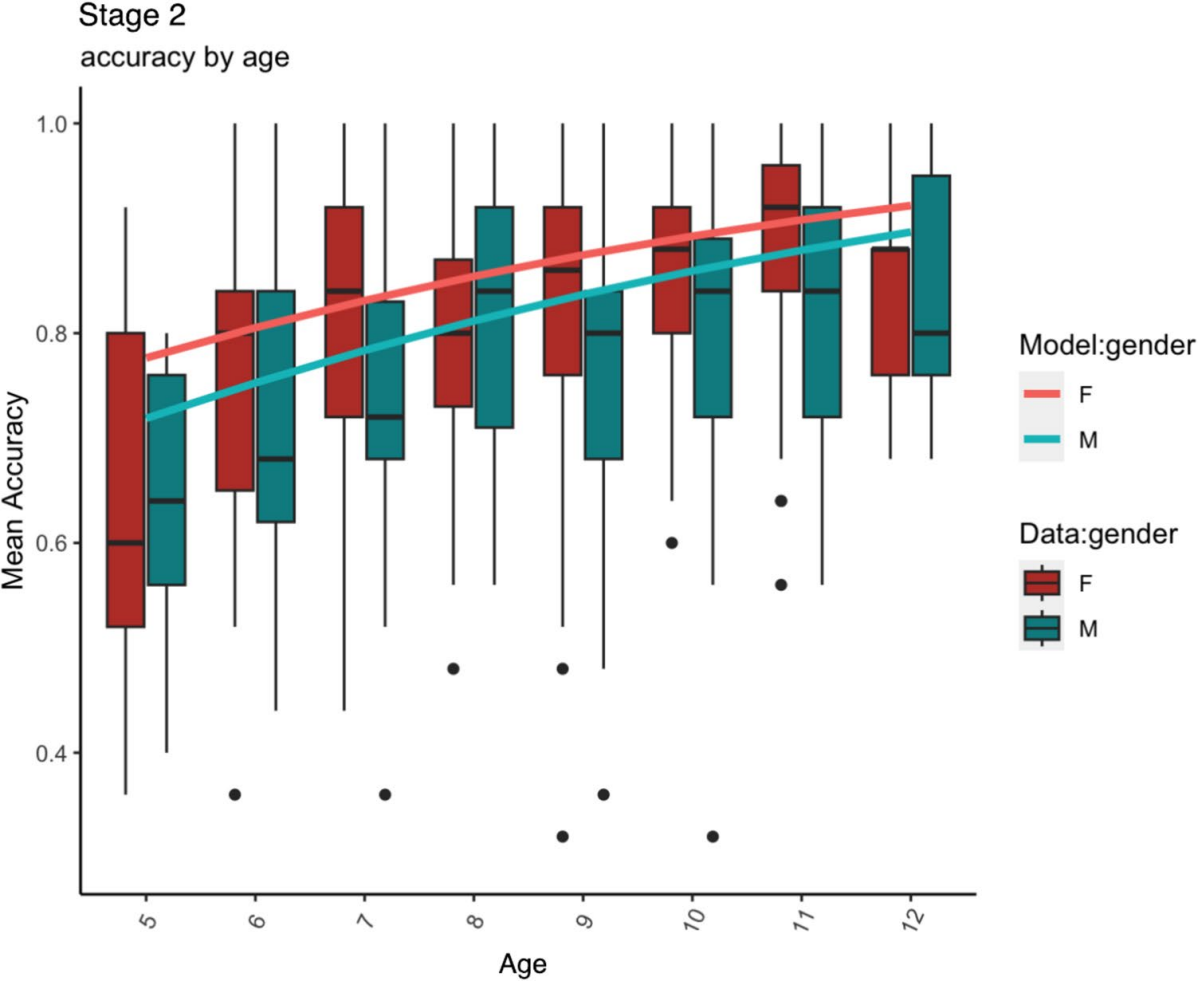
Stage 2 (**no noise**): Mean accuracy data (box plots: *horizontal black lines* indicate medians, the *box* shows the interquartile range and *whiskers* show largest/smallest values within 1.5 times interquartile range above 75^th^/below 25^th^ percentile, *black filled circles* show outliers) and model predictions (***line plots***)

**Fig. 3:**
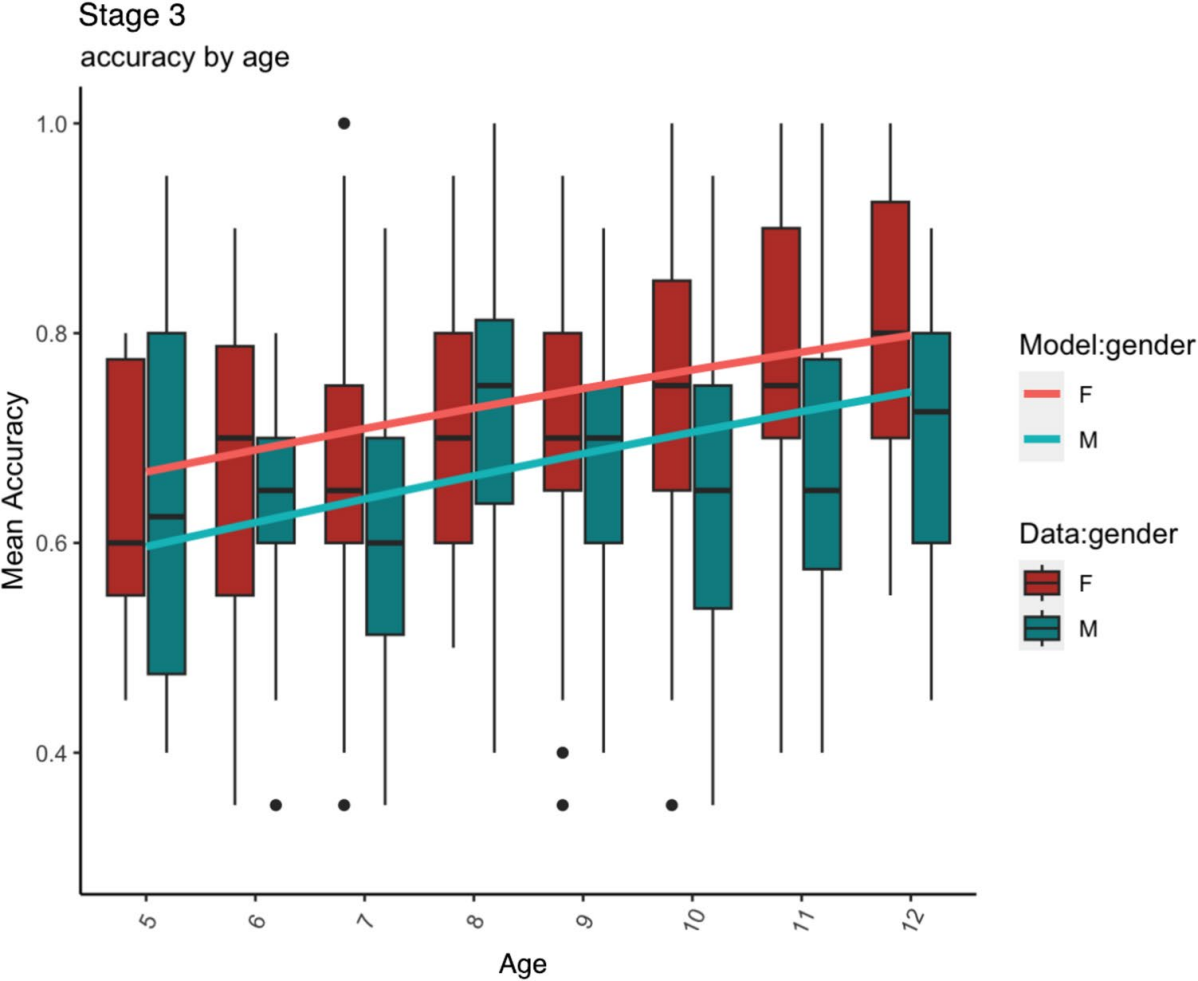
Stage 3. (**Noise**): Mean accuracy data (box plots: *horizontal black lines* indicate medians, the *box* shows the interquartile range and *whiskers* show largest/smallest values within 1.5 times interquartile range above 75^th^/below 25^th^ percentile, *black filled circles* show outliers) and model predictions (**line plots**)

In Figs. 1, 2 and 3, we show, separately for each stage, the individual participant data alongside the corresponding model predictions. In all cases, performance generally improves with age, but it plateaus earlier for Stage 1 compared to Stage 2. The main effect of gender is also clearly visible in all three figures, with girls generally performing better than boys. Stage 1 performance was higher than in the other two (with accuracy in Stage 2 on average 11% lower than in Stage 1). Releveling demonstrated that Stage 2 also had significantly higher performance than Stage 3 (accuracy estimate: – 0.13, logit estimate: – 0.82, SE = 0.21, *p* = .0001; i.e. on average 13% lower accuracy in Stage 3 compared to Stage 2).

**Fig. 4:**
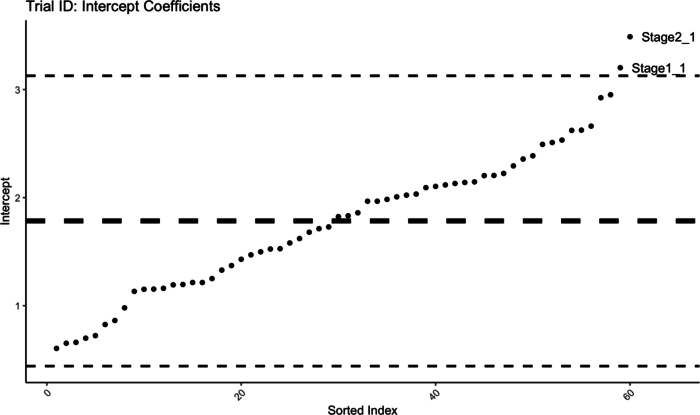
Coefficients for random effect of trials, sorted by magnitude. *Horizontal lines* show mean and mean ± 2 SD. Outlier labels indicate the trial’s stage followed by the index within the stage

Finally, we inspected the random effects coefficients calculated by the model fit (on data from all three stages) to explore whether the CFMT-C task contains any individual trials for which performance differs drastically. A coefficient magnitude diverging far from the average would indicate that the data from this trial (across the whole data set) differed from the rest in a systematic way, which the model-fitting process ‘compensates’ for by increasing the coefficient for this individual trial. Figure 4 shows item coefficients arranged by magnitude. All coefficients fell within 2 SD of the mean, except the first trial in Stage 1 and Stage 2, respectively. That the first trials in a new section would lead to a higher number of errors is, however, unsurprising, and in particular, the first trials in those two stages introduce a new task (first overall trial and first trial where the target is non-identical), whereas the task in Stage 3, while harder due to the addition of Gaussian noise, is similar to Stage 2. Overall it is therefore reassuring to confirm that there are no outliers of concern across the experimental trials.

## Discussion

We set out to characterise age-related differences in face identity recognition abilities present in 5- to 12-year-olds as estimated by one of the most widely used measures of face identity recognition for children: the CFMT-C. With a large sample and detailed approach to data analysis, our investigation yielded new insights into the development of face identity recognition abilities during middle childhood.

We broadly replicate the profile of age-related improvements in recognition performance reported in the only prior investigation of this measure (Croydon et al., 2014). This developmental effect across the targeted age range was evident in the results of a basic linear regression analysis, as well as those of an ANOVA that contrasted the individual year groups represented in our cohort. Critically, this finding was further confirmed with novel linear mixed effects modelling, which allowed us to account for variability across children, and across individual stimuli, in addition to more typically analysed fixed effects. These results contribute important independent support for the conclusion that total scores on the CFMT-C are sensitive to age-related differences in face recognition ability present between the ages of 5 to 12 years (i.e. avoids floor or ceiling effects in this range when considering the complete task). In our sample, performance levels reached ceiling only for the 12-year-olds (the oldest age group), in the first of the three stages (the easiest).

We also included Task Stage (1: Intro, 2: No noise, 3: Noise) as a factor in the linear mixed effects model in order to investigate whether these different components of the CFMT-C task are differentially informative about developmental effects. In line with their increasingly complex demands, participants were confirmed to have performed significantly better in the early compared to the more challenging later stages of the task. Moreover, critically, we identified novel evidence that age-related differences in performance are significantly more pronounced in Stages 2 and 3 compared with Stage 1. In these later conditions, presenting novel images of the test identities forced participants to move beyond simple ‘image recognition’ and more directly encode/retrieve representations of identity. Thus, our results may indicate that we risk underestimating age-related differences when tasks do not present participants with the challenges of real-world face recognition. Future researchers should note that here, we probed performance only with highly controlled stimuli, and developmental differences could be amplified even further when assessed with more naturalistically varying/ambient images (see Zhou et al., 2022). Our motivation to explore whether developmental effects might present differently across the three stages of the task stemmed partly from recent suggestions that Stage 3 of the adult CFMT is of limited utility in certain experimental contexts (see Corrow et al., 2018; Murray & Bate, 2020). Obviously, the benefits of finding ways to streamline the administration of standardised tests are keenly felt in work with populations with limited cognitive resources (including children). Yet, our modelling leads us to conclude that there is no evidence that any stage of the task lacks sensitivity to age-related differences in ability during the middle childhood years.

For the first time, we have identified clear gender differences in performance on the CFMT-C. In line with findings from male and female adults completing the classic adult form CFMT (e.g. Wilmer et al., 2012), girls significantly outperformed boys. This early female advantage in identity recognition adds to broader reports of superior face processing in girls (see Herlitz & Lovén, 2013 for meta-analysis). Our analysis of CFMT-C data did not yield clear evidence about whether the gender bias changes across developmental time, e.g. perhaps becoming amplified through differences in socialisation and experience (see Østergaard et al., 2021). The effect of gender did not interact significantly with age in the best-fitting model of the overall data, which supports the notion that the bias and its underlying mechanism/s are stable between 5 and 12 years of age.

The CFMT-C measures children’s recognition of unfamiliar, white, young adult, male faces. Although these stimuli did not fully constitute a ‘in group’ for any subset of our participants, the restricted nature of the stimuli is far from ideal. Future research could helpfully establish whether the participant characteristics investigated here might also interact with shared vs non-shared characteristics of the stimuli. Such utilisation of a diverse set of faces could be of value across and also within tasks, given that gender differences – for example – may present differently when faces that appear male and female are intermixed (see Herlitz and Lovén, 2013). Given that the CFMT-C comprises only white faces, it is possible that “own ethnicity effects” could have functioned to relatively attenuate perceptual ability for faces from unfamiliar backgrounds (Meissner & Brigham, 2001) and introduced systematic differences in performance among white children and our participant category comprising all other ethnic groups combined. We tested empirically whether any such biases obscured the developmental effects observed in our large, non-segregated sample. Critically, we found that the addition of ethnicity did not improve the fit of our linear mixed model as a main effect or interaction term. Thus, we conclude that the CFMT-C is sufficiently sensitive to robustly characterise group (age, gender) and individual differences in community samples that include individuals from different ethnicities. It is possible, of course, that more pronounced effects of ethnicity might be observed in other testing contexts – e.g. where individuals have had very limited exposure to faces from other backgrounds. However, in our cohort comprising individuals attending school and/or visiting a museum in a large and diverse UK city, these effects did not account for a significant amount of variance in performance on the CFMT-C.

We also found that experimental testing context did not significantly improve our modelling of the CFMT-C scores. Even though participants’ responses were collected as part of seven different studies, which took place in diverse settings, we found that considering this variability did not meaningfully affect the pattern of observed results. This finding is encouraging for developmental researchers like ourselves who are motivated to find creative solutions when addressing the challenge of collecting representative, large-scale developmental data sets. Such work benefits from being able to move outside the lab into community settings, which has been considered to be associated with some loss of desirable experimental control. Crucially, however, the current findings support the robustness of the CFMT-C across diverse testing contexts, as well as the generalisability of the current results.

Inspecting the random effects coefficients for individual trials in the best-fitting multilevel model further demonstrated that there are no unexplained outliers, with all coefficients within 2 standard deviations of the overall mean magnitude. The only trials with unexpectedly large coefficients, which indicates that the pattern of responses was different compared to other trials, were the first trials in Stage 1 and Stage 2, respectively. Naturally, the very first trial and the first trial in a changed task scenario are likely to incur a higher error rate than the remaining trials. This further confirms the suitability of the CFMT in its current form for investigating children’s face recognition skills. Together, the results of the current study replicate and extend the previous psychometric examination of the CFMT-C, and highlight the value of applying multilevel statistical models to characterise the factors driving performance variability. We confirm not only that there are age-related improvements in performance on the task, but that all three stages of the measure are informative regarding these developmental effects – with the latter, more difficult stages, proving the most sensitive.

The reliability of the observed individual differences in children’s face processing will be an interesting avenue for future research, as will the selectivity of strengths and weaknesses in this domain. Having measured only face perception, our results cannot speak to the extent to which the observed changes in recognition ability and its underlying processes are face selective vs more general (a subject of ongoing debate, see McKone et al., 2012 for review). Still, for the first time we have clearly identified gender differences in performance on this widely used measure of face identity recognition, which aligns with those observed on other measures and with other age groups (Herlitz & Lovén, 2013) and is not observed for all object categories (see McGugin et al., 2012).

We establish empirically that the CFMT-C is robust across participant ethnicity groups and testing environments (when administered by the same testing group). It is also robust across items – that is, there are no individual trials yielding aberrant levels of performance, which further speaks to the empirical quality of the measure. Thus, we can broadly conclude that the CFMT-C is an extremely useful tool for researchers interested in group and individual differences in face identity recognition ability during the childhood years.

## Supplementary information

Below is the link to the electronic supplementary material.

Supplementary file1 (DOCX 19 KB)

## Data Availability

The CFMT-C is freely available for researchers assessing children’s face memory and can be downloaded here https://ccd.edu.au/engagement-resources/resources-and-tools/cfmtfc/. The deidentified data set from the current study is available at https://osf.io/rk7eh/?view_only=5a84e14c1c134d10bc3ec42d0429a19e and this experiment was not preregistered.

## Footnotes

1 In addition, this approach eliminates issues with assumptions around heteroscedasticity inherent to the analysis of categorical data using ANOVAs (Agresti, 2002; Dixon, 2008; Jaeger, 2008) which may result in poorer characterisation of the data.

2 Ethnicity data is not available for 113 participants.

3 $$\text{glmer}\left(\text{Accuracy}\sim\text{Age}+\text{Gender}+\text{Stage}+\text{Age}:\text{Stage}+\left(1\left\vert{\text{Trial}}_\text{ID}\right.\right)+\left(\left.\text{Stage}\right\vert\text{subject}\right),\text{data}=\text{Data},\text{family}={^\prime}{^\prime}{\text{binomial}{^\prime}{^\prime}}\right)$$  

## Code availability

The analysis code for this experiment is available at https://osf.io/rk7eh/?view_only=5a84e14c1c134d10bc3ec42d0429a19e.

## References

[CR1] Agresti, A. (2002). *Categorical Data Analysis* (2nd ed.). Wiley.

[CR2] Arrington, M., Elbich, D., Dai, J., Duchaine, B., & Scherf, K. S. (2022). Introducing the female Cambridge Face Memory Test–long form (F-CFMT+). *Behavior**Research Methods*, *1–14*. 10.3758/s13428-022-01805-8 10.3758/s13428-022-01805-8 PMC8863095 35194750

[CR3] Barr, D. J., Levy, R., Scheepers, C., & Tily, H. J. (2013). Random effects structure for confirmatory hypothesis testing: Keep it maximal. *Journal of Memory and Language,**68*, 255–278. 10.1016/j.jml.2012.11.001 10.1016/j.jml.2012.11.001 PMC3881361 24403724

[CR4] Bates, D., Mächler, M., Bolker, B., & Walker, S. (2015). Fitting linear mixed-effects models using lme4. *Journal of Statistical Software,**67*(1), 1–48. 10.18637/jss.v067.i01

[CR5] Bennetts, R. J., Murray, E., Boyce, T., & Bate, S. (2017). Prevalence of face recognition deficits in middle childhood. *Quarterly Journal of Experimental Psychology,**70*(2), 234–258. 10.1080/17470218.2016.1167924 10.1080/17470218.2016.1167924 26999413

[CR6] Benton, A. L., & Van Allen, M. W. (1968). Impairment in facial recognition in patients with cerebral disease. *Cortex,**4*(4), 344-IN1. 5711050

[CR7] Boutet, I., & Meinhardt-Injac, B. (2021). Measurement of individual differences in face-identity processing abilities in older adults. *Cognitive Research: Principles and Implications,**6*(1), 1–11. 10.1186/s41235-021-00310-4 34275050 10.1186/s41235-021-00310-4 PMC8286909

[CR8] Bowles, D. C., McKone, E., Dawel, A., Duchaine, B., Palermo, R., Schmalzl, L., ..., & Yovel, G. (2009). Diagnosing prosopagnosia: Effects of ageing, sex, and participant–stimulus ethnic match on the Cambridge Face Memory Test and Cambridge Face Perception Test. *Cognitive Neuropsychology*, *26*(5), 423–455. 10.1080/02643290903343149 10.1080/02643290903343149 19921582

[CR9] Childs, M. J., Jones, A., Thwaites, P., Zdravković, S., Thorley, C., Suzuki, A., & Tree, J. J. (2021). Do individual differences in face recognition ability moderate the other ethnicity effect? *Journal of Experimental Psychology: Human Perception and Performance,**47*(7), 893. 10.1037/xhp0000762 34292047 10.1037/xhp0000762

[CR10] Cho, S. J., Wilmer, J., Herzmann, G., McGugin, R. W., Fiset, D., Van Gulick, A. E., ..., & Gauthier, I. (2015). Item response theory analyses of the Cambridge Face Memory Test (CFMT). *Psychological Assessment*, *27*(2), 552. 10.1037/pas0000068 10.1037/pas0000068 PMC4461534 25642930

[CR11] Corrow, S. L., Albonico, A., & Barton, J. J. (2018). Diagnosing prosopagnosia: The utility of visual noise in the Cambridge Face Recognition Test. *Perception,**47*(3), 330–343. 10.1177/0301006617750045 29320938 10.1177/0301006617750045

[CR12] Croydon, A., Pimperton, H., Ewing, L., Duchaine, B. C., & Pellicano, E. (2014). The Cambridge Face Memory Test for Children (CFMT-C): A new tool for measuring face recognition skills in childhood. *Neuropsychologia,**62*, 60–67. 10.1016/j.neuropsychologia.2014.07.008 25054837 10.1016/j.neuropsychologia.2014.07.008

[CR13] Curran, P. J., & Hussong, A. M. (2009). Integrative data analysis: The simultaneous analysis of multiple data sets. *Psychological Methods,**14*(2), 81. 10.1037/a0015914 19485623 10.1037/a0015914 PMC2777640

[CR14] Dalrymple, K. A., Garrido, L., & Duchaine, B. (2014). Dissociation between face perception and face memory in adults, but not children, with developmental prosopagnosia. *Developmental Cognitive Neuroscience,**10*, 10–20. 10.1016/j.dcn.2014.07.003 25160676 10.1016/j.dcn.2014.07.003 PMC6987906

[CR15] Dennett, H. W., McKone, E., Tavashmi, R., Hall, A., Pidcock, M., Edwards, M., & Duchaine, B. (2012). The Cambridge Car Memory Test: A task matched in format to the Cambridge Face Memory Test, with norms, reliability, sex differences, dissociations from face memory, and expertise effects. *Behavior Research Methods,**44*(2), 587–605. 10.3758/s13428-011-0160-2 22012343 10.3758/s13428-011-0160-2

[CR16] Dixon, P. (2008). Models of accuracy in repeated-measures designs. *Journal of Memory and Language,**59*, 447–456. 10.1016/j.jml.2007.11.004

[CR17] Duchaine, B., & Nakayama, K. (2006). The Cambridge Face Memory Test: Results for neurologically intact individuals and an investigation of its validity using inverted face stimuli and prosopagnosic participants. *Neuropsychologia,**44*(4), 576–585. 10.1016/j.neuropsychologia.2005.07.001 16169565 10.1016/j.neuropsychologia.2005.07.001

[CR18] Ewing, L., Pellicano, E., King, H., Lennuyeux-Comnene, L., Farran, E. K., Karmiloff-Smith, A., & Smith, M. L. (2018). Atypical information-use in children with autism spectrum disorder during judgments of child and adult face identity. *Developmental Neuropsychology,**43*(4), 370–384. 10.1080/87565641.2018.1449846 29558171 10.1080/87565641.2018.1449846 PMC5964451

[CR19] Ewing, L., Mares, I., Edwards, S. G., & Smith, M. L. (2022). Orientation effects support specialist processing of upright unfamiliar faces in children and adults. *Developmental Psychology,**59*(6), 1109–1115. 10.1037/dev0001454 36095246 10.1037/dev0001454

[CR20] Farran, E. K., Mares, I., Papasavva, M., Smith, F. W., Ewing, L., & Smith, M. L. (2020). Characterizing the neural signature of face processing in Williams syndrome via multivariate pattern analysis and event-related potentials. *Neuropsychologia,**142*, 107440. 32179101 10.1016/j.neuropsychologia.2020.107440

[CR21] Germine, L. T., Duchaine, B., & Nakayama, K. (2011). Where cognitive development and aging meet: Face learning ability peaks after age 30. *Cognition,**118*(2), 201–210. 10.1016/j.cognition.2010.11.002 21130422 10.1016/j.cognition.2010.11.002

[CR22] Herlitz, A., & Lovén, J. (2013). Sex differences and the own-gender bias in face recognition: A meta-analytic review. *Visual Cognition.,**21*, 1306–36. 10.1080/13506285.2013.823140

[CR23] Herzmann, G., Danthiir, V., Schacht, A., Sommer, W., & Wilhelm, O. (2008). Toward a comprehensive test battery for face cognition: Assessment of the tasks. *Behavior Research Methods,**40*, 840–857. 10.3758/BRM.40.3.840 18697680 10.3758/brm.40.3.840

[CR24] Jaeger, T. F. (2008). Categorical data analysis: Away from ANOVAs (transformation or not) and towards logit mixed models. *Journal of Memory and Language,**59*, 434–446. 10.1016/j.jml.2007.11.007 19884961 10.1016/j.jml.2007.11.007 PMC2613284

[CR25] Kho, S. K., Leong, B. Q. Z., Keeble, D. R., Wong, H. K., & Estudillo, A. J. (2023). A new Asian version of the CFMT: The Cambridge Face Memory Test–Chinese Malaysian (CFMT-MY). *Behavior**Research Methods*, 1–15. 10.3758/s13428-023-02085-6 10.3758/s13428-023-02085-6 36971958

[CR26] Mares, I., Ewing, L., Farran, E. K., Smith, F. W., & Smith, M. L. (2020). Developmental changes in the processing of faces as revealed by EEG decoding. NeuroImage, *211* (September 2019). 10.1016/j.neuroimage.2020.116660 10.1016/j.neuroimage.2020.116660 32081784

[CR27] Mares, I., Ewing, L., Papasavva, M., Ducrocq, E., Smith, F. W., & Smith, M. L. (2023). Face recognition ability is manifest in early dynamic decoding of face-orientation selectivity—Evidence from multi-variate pattern analysis of the neural response. *Cortex,**159*, 299–312. 10.1016/j.cortex.2022.11.004 36669447 10.1016/j.cortex.2022.11.004

[CR28] McGugin, R. W., Richler, J. J., Herzmann, G., Speegle, M., & Gauthier, I. (2012). The vanderbilt expertise test reveals domain-general and domain-specific sex effects in object recognition. *Vision Research,**69*, 10–22. 22877929 10.1016/j.visres.2012.07.014 PMC3513270

[CR29] McGugin, R. W., Van Gulick, A. E., & Gauthier, I. (2016). Cortical thickness in fusiform face area predicts face and object recognition performance. *Journal of Cognitive Neuroscience,**28*(2), 282–294. 10.1162/jocn_a_00891 26439272 10.1162/jocn_a_00891 PMC5034353

[CR30] McKone, E., Hall, A., Pidcock, M., Palermo, R., Wilkinson, R. B., Rivolta, D., ..., & O’Connor, K. B. (2011). Face ethnicity and measurement reliability affect face recognition performance in developmental prosopagnosia: Evidence from the Cambridge Face Memory Test–Australian. *Cognitive Neuropsychology*, *28*(2), 109–146. 10.1080/02643294.2011.616880 10.1080/02643294.2011.616880 22122116

[CR31] McKone, E., Crookes, K., Jeffery, L., & Dilks, D. D. (2012). A critical review of the development of face recognition: Experience is less important than previously believed. *Cognitive Neuropsychology,**29*(1–2), 174–212. 10.1080/02643294.2012.660138 22360676 10.1080/02643294.2012.660138

[CR32] McKone, E., Wan, L., Robbins, R., Crookes, K., & Liu, J. (2017). Diagnosing prosopagnosia in East Asian individuals: Norms for the Cambridge Face Memory Test-Chinese. *Cognitive Neuropsychology,**34*(5), 253–268. 10.1080/02643294.2017.1371682 28906173 10.1080/02643294.2017.1371682

[CR33] Meissner, C. A., & Brigham, J. C. (2001). Thirty years of investigating the own-race bias in memory for faces: A meta-analytic review. *Psychology, Public Policy, and Law,**7*(1), 3. 10.1037/1076-8971.7.1.3

[CR34] Murray, E., & Bate, S. (2020). Diagnosing developmental prosopagnosia: Repeat assessment using the Cambridge Face Memory Test. *Royal Society Open Science,**7*(9), 200884. 10.1098/rsos.200884 33047048 10.1098/rsos.200884 PMC7540801

[CR35] O’Hearn, K., Schroer, E., Minshew, N., & Luna, B. (2010). Lack of developmental improvement on a face memory task during adolescence in autism. *Neuropsychologia,**48*(13), 3955–3960. 10.1016/j.neuropsychologia.2010.08.024 20813119 10.1016/j.neuropsychologia.2010.08.024 PMC2975893

[CR36] Østergaard Knudsen, C., Winther Rasmussen, K., & Gerlach, C. (2021). Gender differences in face recognition: The role of holistic processing. *Visual Cognition,**29*(6), 379–385.

[CR37] Rhodes, G., Jeffery, L., Taylor, L., Hayward, W. G., & Ewing, L. (2014). Individual differences in adaptive coding of face identity are linked to individual differences in face recognition ability. *Journal of Experimental Psychology: Human Perception and Performance,**40*(3), 897. 10.1037/a0035939 24684315 10.1037/a0035939

[CR38] Richler, J. J., Cheung, O. S., & Gauthier, I. (2011). Holistic processing predicts face recognition. *Psychological Science,**22*(4), 464–471. 10.1177/095679761140175 21393576 10.1177/0956797611401753 PMC3077885

[CR39] Russell, R., Duchaine, B., & Nakayama, K. (2009). Super-recognizers: People with extraordinary face recognition ability. *Psychonomic Bulletin & Review,**16*(2), 252–257. 10.3758/PBR.16.2.252 10.3758/PBR.16.2.252 PMC3904192 19293090

[CR40] Warrington, E. K. (1984). *Recognition Memory Test: Manual*. NFER-Nelson.

[CR41] Wilmer, J. B. (2017). Individual differences in face recognition: A decade of discovery. *Current Directions in Psychological Science,**26*(3), 225–230. 10.1177/0963721417710693

[CR42] Wilmer, J. B., Germine, L., Chabris, C. F., Chatterjee, G., Gerbasi, M., & Nakayama, K. (2012). Capturing specific abilities as a window into human individuality: The example of face recognition. *Cognitive Neuropsychology,**29*(5–6), 360–392. 10.1080/02643294.2012.753433 23428079 10.1080/02643294.2012.753433 PMC3630451

[CR43] Zeileis, A., & Hothorn, T. (2002). Diagnostic checking in regression relationships. *R News,**2*(3), 7–10.

[CR44] Zhou, X., Vyas, S., Ning, J., & Moulson, M. C. (2022). Naturalistic face learning in infants and adults. *Psychological Science,**33*(1), 135–151. 34919451 10.1177/09567976211030630 PMC13038100

