# Supplementary material for: Exploring the development of face recognition across childhood via logistic mixed-effects modelling of the standardised Cambridge Face Memory Test
Source: Behav Res Methods. 2025 Mar 10;57(4):113. doi: 10.3758/s13428-025-02629-y (PMC11893692; doi:10.3758/s13428-025-02629-y)
Supplement: Supplementary file 1 — Supplementary file1 (DOCX 19 KB) [file 13428_2025_2629_MOESM1_ESM.docx]

Supplementary Materials

Table 1.

| Exp/Age | Testing Context | **5** | **6** | **7** | **8** | **9** | **10** | **11** | **12** |
| --- | --- | --- | --- | --- | --- | --- | --- | --- | --- |
| **A** | ‘Holiday camp’ setting | F:6/M:0 | 2/2 | 7/1 | 4/2 | 5/0 | 2/3 | 3/1 | 1/0 |
| **B** | Lab based study 1 | 2/0 | 2/0 | 8/1 | 4/2 | 4/3 | 2/4 | 2/2 | 2/1 |
| **C** | Lab based study 2 | 0/0 | 2/2 | 7/6 | 5/0 | 4/4 | 2/2 | 4/0 | 1/3 |
| **D** | Lab based study 3 | 0/0 | 0/0 | 1/1 | 3/3 | 4/4 | 1/1 | 0/0 | 0/0 |
| **E** | Science Museum | 7/4 | 15/8 | 20/9 | 18/11 | 23/15 | 13/17 | 14/17 | 12/6 |
| **F** | Schools testing 1 | 0/1 | 9/4 | 11/13 | 8/9 | 10/6 | 16/9 | 10/9 | 0/0 |
| **G** | Schools testing 2 | 0/0 | 12/8 | 23/19 | 4/5 | 24/13 | 10/9 | 8/10 | 0/0 |

Note: Sample characteristics across the different testing contexts for the CFMT-C (7-level between-subjects factor in the analyses). Participant numbers for Experiments A-G by age group and gender. For each cell the first number refers to the number of females, the second number to the number of males.

Table 2.

| Effect added | Log Likelihood | DF | Chi Square | p |
| --- | --- | --- | --- | --- |
| (Base) | -14971 |  |  |  |
| (1\|TrialID) | -13370 | 1 | 3202.6652 | <.0001 |
| (1\|ParticipantID) | -12792 | 1 | 1154.7643 | <.0001 |
| Age | -12765 | 1 | 54.3588 | <.0001 |
| Gender | -12750 | 1 | 29.2461 | <.0001 |
| Age:Gender | -12748 | 1 | 3.6905 | 0.05472 |
| Ethnicity | -12748 | 1 | 0.2908 | 0.58972 |
| Age:Ethnicity | -12748 | 1 | 1.4537 | 0.22794 |
| Gender:Ethnicity | -12748 | 1 | 0.3013 | 0.58305 |
| Age:Gender:Ethnicity | -12747 | 1 | 0.2712 | 0.60251 |
| Stage | -12721 | 2 | 52.0914 | <.0001 |
| (Stage\|Subject) | -12673 | 5 | 96.193 | <.0001 |
| Age:Stage | -12652 | 2 | 42.3308 | <.0001 |
| Gender:Stage | -12651 | 2 | 1.2026 | 0.54809 |
| Ethnicity:Stage | -12651 | 2 | 1.6293 | 0.44278 |
| Age:Gender:Stage | -12650 | 2 | 1.1373 | 0.56629 |
| Age:Ethnicity:Stage | -12650 | 2 | 0.538 | 0.76415 |
| Gender:Ethnicity:Stage | -12649 | 2 | 0.7877 | 0.67445 |
| Age:Gender:Ethnicity:Stage | -12648 | 2 | 2.872 | 0.23788 |

Note: Model comparisons for those 494 participants where ethnicity information was available

Table 3.

| Added Effect | LogLik | Df | Chisq | Pr(>Chisq) |
| --- | --- | --- | --- | --- |
| (Base) | -18418 |  |  |  |
| (1\|TrialID) | -16466 | 1 | 3905.4752 | <.0001 |
| {1\|ParticipantID) | -15736 | 1 | 1458.2994 | <.0001 |
| Age | -15705 | 1 | 62.8348 | <.0001 |
| Gender | -15695 | 1 | 19.8794 | <.0001 |
| Age:Gender | -15694 | 1 | 2.9452 | 0.08614 |
| Stage | -15667 | 2 | 52.7947 | <.0001 |
| (Stage\|ParticipantID) | -15590 | 5 | 155.0317 | <.0001 |
| Age:Stage | -15564 | 2 | 51.4143 | <.0001 |
| Gender:Stage | -15563 | 2 | 1.5619 | 0.45798 |
| Age:Gender:Stage | -15562 | 2 | 2.2496 | 0.32472 |

Note: Main model comparison with 607 participants, before removal of non-significant effects

Table 4

| Added Effect | Log Likelihood | DF | ChiSquare | p |
| --- | --- | --- | --- | --- |
| Base | -18418 |  |  |  |
| (1\|TrialID) | -16466 | 1 | 3905.475 | <.0001 |
| {1\|ParticipantID) | -15736 | 1 | 1458.299 | <.0001 |
| Age | -15705 | 1 | 62.835 | <.0001 |
| Gender | -15695 | 1 | 19.879 | <.0001 |
| Stage | -15669 | 2 | 52.796 | <.0001 |
| (Stage\|ParticipantID) | -15591 | 5 | 155.182 | <.0001 |
| Age:Stage | -15565 | 2 | 51.647 | <.0001 |

Note: Main model comparison with 607 participants, after removal of non-significant effects

Table 5

| Effect added | Log Likelihood | DF | ChiSquare | p |
| --- | --- | --- | --- | --- |
| (Best Model) | -15565 |  |  |  |
| Experiment | -15561 | 6 | 8.47 | 0.21 |
| Experiment:Age | -15556 | 6 | 9.3672 | 0.15 |
| Experiment:Gender | -15551 | 6 | 11.0705 | 0.09 |
| Experiment:Stage | -15541 | 12 | 19.3405 | 0.08 |

Note: Model comparisons for main effects and interactions of “Experiment” (levels:A-G)
